# Data-Driven Modeling and Response Prediction of Cut-Out Type Piezoelectric Beams

**DOI:** 10.3390/mi17040450

**Published:** 2026-04-06

**Authors:** Mingli Bian, Wenan Jiang, Qinsheng Bi

**Affiliations:** Faculty of Civil Engineering and Mechanics, Jiangsu University, Zhenjiang 212013, China; bianmingli1223@163.com (M.B.); qshengbi@ujs.edu.cn (Q.B.)

**Keywords:** BP neural network, limiter, piezoelectric energy harvesting, nonlinear, response prediction

## Abstract

In addressing the issue of insufficient theoretical model accuracy for Cut-out type piezoelectric beams with limiters under the influence of contact-impact nonlinearity, this study utilizes the backpropagation neural network algorithm to develop a data-driven modeling approach based on experimental data from partial distance parameters. This approach aims to achieve accurate predictions of the output voltage and displacement responses of the energy harvester. For different parameter combinations of the limiter gap distance *d* and installation distance *a*, amplitude–frequency response data were first systematically collected through experiments, along with time–voltage response data corresponding to different load resistances. Using these data, a training sample set was constructed, and a multi-layer BP neural network prediction model was established with frequency or time as the input and voltage and displacement responses as the outputs. Validation against experimental data demonstrated that the BP neural network can accurately extrapolate and predict the amplitude–frequency response curves of voltage and displacement under various distance parameter combinations, as well as accurately predict the transient voltage outputs under different load conditions.

## 1. Introduction

Against the backdrop of the rapid development of technologies such as the Internet of Things (IoT), Micro-Electro-Mechanical Systems (MEMSs), and portable electronic devices, achieving self-powering and sustained stable operation for these devices has become a crucial research objective. Efficiently harvesting and utilizing vibration energy from the surrounding environment has thus emerged as a significant research focus in academia [1,2,3,4,5,6,7]. Among various types of energy harvesters, piezoelectric vibration energy harvesters have attracted extensive attention from researchers due to their advantages of simple structure, high power density, lack of electromagnetic interference, and ease of integration and miniaturization [8,9,10].

**Figure 1:**
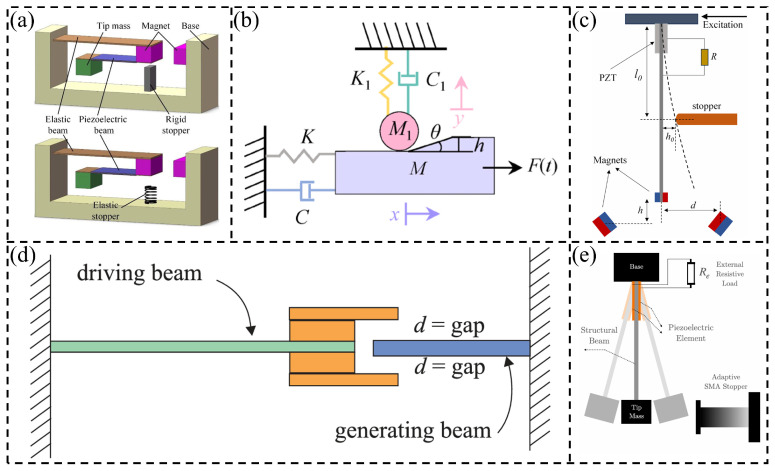
Schematic diagrams of multi-type limiter models for piezoelectric vibration energy harvesters: (**a**) limiters with different stiffnesses [25]; (**b**) stepped limiter [26]; (**c**) unilateral vertically movable limiter [27]; (**d**) C-shaped limiter [28]; (**e**) adaptive non-smooth limiter based on shape memory alloy [29].

Traditional linear piezoelectric vibration energy harvesters suffer from narrow-band frequency response, making it difficult to efficiently harvest energy from broadband environmental vibrations [11,12]. To address this issue, introducing nonlinear factors, such as magnetic coupling [13,14] or mechanical impact [15], can broaden the effective operating bandwidth of the harvester. A typical strategy involves installing displacement limiters on linear piezoelectric beams, which introduces nonlinearity efficiently through contact-impact during large-amplitude vibrations, thereby enhancing both energy harvesting efficiency and power output density [16]. Liu et al. [17] demonstrated as early as 2012 that mechanical limiters could be used to expand the operational bandwidth of an energy harvester, extending it from 30 Hz to 48 Hz. Subsequently, in 2018, Hu et al. [18] developed a two-degree-of-freedom (2-DOF) piezoelectric energy harvester featuring a special mechanical limiting device. Through analytical modeling and numerical simulation, the proposed device is shown to achieve power output improvements of 64.4% and 118.9% compared to linear single-degree-of-freedom (SDOF) and 2-DOF systems, respectively. Recently, Shao et al. [19] developed an innovative broadband 2-DOF energy harvesting system featuring an integrated motion limiter applied to a folded piezoelectric beam and demonstrated that the incorporation of amplitude limitation effectively modifies the vibration mode of the piezoelectric beam, thereby broadening the operational frequency range. As a result, the system achieves a maximum power output of 713 μW. Jiang et al. [20] showed that incorporating stoppers into a V-shaped harvester broadens the resonance bandwidth. Qi et al. [21] reported a 3.54-fold increase in output power for their harvester with a stopper. Fan et al. [22] developed a nonlinear piezoelectric energy harvester (PEH) array using two pairs of motion limiters; the strong hardening effect resulting from collisions expanded the frequency bandwidth by 240% under parametric and direct excitation. Zhao et al. [23] developed a wind energy harvester with a stopper and demonstrated its promising energy harvesting capability. Maamer et al. [24] highlighted the importance of frequency up-converting technology for harvesting low-frequency vibration energy through the study of impact-driven low-frequency energy harvesters. He et al. [25] proposed a 2-DOF nonlinear piezoelectric vibration energy harvester with limiters, with experimental results showing that rigid limiters achieved a maximum absolute average voltage of 27.56 V at 19.06 Hz. Lin et al. [26] designed and investigated a deformation-limited piezoelectric vibration energy harvester with limiters, shown in Figure 1b. The study found that as the limiter height difference *h* increased from 1 mm to 4 mm, the maximum output voltage significantly increased from 13.6 V to 41.2 V. When the inclination angle *θ* increased from 20° to 50°, the maximum output voltage slightly increased from 22 V to 23.2 V.

Wang et al. [27] proposed a scheme with an adjustable unilateral limiter installed on the side of a deeper potential well, achieving an increase in inter-well vibration proportion from 66.8% to 100% under 0.35 g and 5.9 Hz, as well as raising the inter-well vibration probability from 60.7% to 99.5% as the excitation frequency increased from 2.9 Hz to 9.0 Hz. Machado et al. [28] designed a frequency up-conversion piezoelectric vibration energy harvester based on dual impacts, shown in Figure 1d. Experimental results indicated that a piezoelectric beam with a thickness of 0.4 mm could achieve a peak power as high as 1428 μW at 20 Hz with a gap distance of 3 mm. Adeodato et al. [29] designed an adaptive non-smooth limiter based on shape memory alloy, shown in Figure 1e. The research found that using heating of a shape memory alloy from 271 K to 291 K, the energy harvester achieved a maximum output power of 4.47 μW at the linear resonant frequency ω = 146 rad/s, maintained 3.7 μW at ω = 186.8 rad/s, and doubled the 30 s average output power from 0.7 μW to 1.44 μW compared to a linear harvester. In 2026, Su et al. [30] investigated a 2-DOF impact-driven piezoelectric energy harvester under rotational excitation and found that by incorporating a limiter and using a 90 mm main beam, the device achieved a broadband voltage output of 8~12 Hz with a peak of 2.0 V under a 1 MΩ load, while the two resonant frequencies shifted to 8.6 Hz and 10.5 Hz with a spacing of only 1.9 Hz. Feng et al. [31] developed a nonlinear magnetic-collision piezoelectric energy harvester with lever amplification, integrating a double-sided limiter and magnets to achieve voltage increases of 78.02% and 101.78% compared to configurations without magnets and without a limiter, respectively, and an output power of 56.01 mW at 30 kΩ under optimal parameters.

However, obtaining experimental data is often costly and time-consuming, which severely restricts research efficiency. In this context, data-driven modeling methods, particularly those based on neural network algorithms, offer new perspectives for modeling nonlinear energy harvesting systems. Xia et al. [32] proposed a simulation framework validated by a hybrid deep neural network, achieving a coefficient of determination of 0.984 for power output prediction (RMSE 0.57 W, MAE 0.42 W), 0.972 for impedance characteristic prediction (RMSE 1.22 Ω), and 0.961 for voltage distribution prediction (RMSE 4.73 V), demonstrating accurate capture of nonlinear electromechanical coupling. Martins et al. [33] employed the Non-dominated Sorting Genetic Algorithm-II (NSGA-II) with an artificial neural network (ANN) metamodel requiring only 3000 samples to achieve multi-objective robust optimization for maximizing the mean output power frequency response function and minimizing relative dispersion, completing the optimization in 3 min and 42 s—over 95% faster than the 1 h and 20 min required by the finite element model. Adhikari et al. [34] proposed a solution integrating finite element analysis with machine learning, where a deep neural network (DNN) with Tanh activation function outperformed the traditional ANN model, improving the overall coefficient of determination from 0.97399 to 0.98921 and reducing the mean square error from 1.6851 to 0.37556. Kacimi et al. [35] designed a maximum power point tracking (MPPT) method using a radial basis function (RBF) neural network optimized by particle swarm optimization (PSO). The results show that after optimization during the learning phase, the RBF network requires only six neurons to achieve an energy harvesting efficiency exceeding 99%. He et al. [36] proposed a method based on the coordinated optimization of a backpropagation neural network (BPNN) and NSGA-II. At 75 Hz, this approach increased the peak voltage by 12%, 22%, and 32% for energy harvesters with positive, zero, and negative Poisson’s ratios, respectively.

Currently, modeling the amplitude–frequency response of Cut-out type piezoelectric vibration energy harvesters with limiters is extremely challenging. This is due to the involvement of multi-physics field coupling (contact–structure–electric) and the introduction of strong nonlinearities caused by mechanical impact. Additionally, conducting full parametric domain experiments by varying the gap distance *d* between the Cut-out type piezoelectric beam and the limiter and the distance *a* from the limiter to the fixed end of the beam is not only time-consuming and costly but also makes it difficult to obtain a large volume of experimental data.

Therefore, this paper employs a backpropagation (BP) neural network for data-driven modeling. A training sample set for the BP neural network is constructed using experimental data corresponding to a limited combination of distance parameters *d* and *a*. This approach enables the prediction of the voltage and displacement amplitude–frequency responses of the harvester for extrapolated parameter combinations.

This not only provides an efficient analysis method for predicting the amplitude–frequency response of Cut-out type piezoelectric vibration energy harvesters with limiters, addressing the issue of insufficient accuracy in traditional mechanistic modeling, but also offers a new pathway for performance evaluation and design optimization of such strongly nonlinear piezoelectric vibration energy harvesting systems with contact-impact.

## 2. BP Neural Network Prediction Model

### 2.1. Principle of the Algorithm

The BP neural network belongs to a class of multi-layer feedforward neural networks (FNN), and its training mechanism relies on the fundamental principle of error backpropagation. The BP neural network developed in this work employs seven input neurons in the input layer, corresponding to the limiter gap distance *d* and installation distance *a* between the limiter and the piezoelectric beam, the frequency *f*, and the local features extracted from the voltage or displacement signals—namely, the local mean $\mu_{local}$, local standard deviation $\sigma_{local}$, gradient ∇V, and curvature $\nabla^{2}V$. The normalized voltage or displacement value serves as the output.

**Figure 2:**
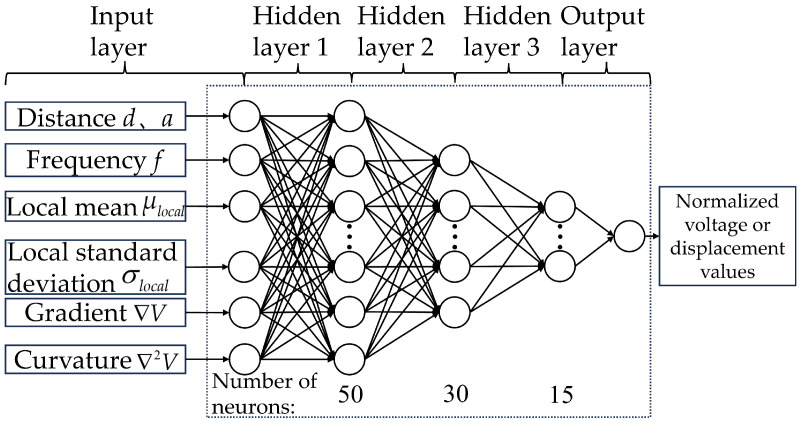
Topology of the BP neural network for predicting voltage and displacement amplitude–frequency responses.

Figure 2 illustrates the topology of this BP neural network. As shown in Figure 2, the number of neurons in the three hidden layers of this BP neural network is 50, 30, and 15, respectively.

During the forward propagation from the input layer to the first hidden layer, the *j*-th hidden layer neuron satisfies the following relationship:

(1) $$\left\{\begin{array}{l} z_{j}^{\left(1\right)}=\sum_{i=1}^{6}w_{ji}^{\left(1\right)}x_{i}+b_{j}^{\left(1\right)}\\ a_{j}^{\left(1\right)}=f(z_{j}^{\left(1\right)}) \end{array}\right.$$

Among these, $z_{j}^{\left(1\right)}$, $b_{j}^{\left(1\right)}$, and $a_{j}^{\left(1\right)}$ represent the weighted input, bias term, and output of the *j*-th neuron in the first hidden layer, respectively; $w_{ji}^{\left(1\right)}$ denotes the weight from the input layer to the first hidden layer; and f(∙) refers to the activation function. It should be noted that the activation function f(∙) used in this BP neural network algorithm is the hyperbolic tangent function

(2) $$f(z)=tanh(z)=\frac{e^{z}-e^{-z}}{e^{z}+e^{-z}}$$

The propagation between the *l*-th hidden layer and the (*l* + 1)-th hidden layer can be expressed as

(3) $$\left\{\begin{array}{l} z_{k}^{\left(l+1\right)}=\sum_{j=1}^{n_{l}}w_{\mathrm{kj}}^{\left(l+1\right)}a_{j}^{\left(l\right)}+b_{k}^{\left(l+1\right)}\\ a_{k}^{\left(l+1\right)}=f(z_{k}^{\left(l+1\right)}) \end{array}\right.$$

Here, *l* and *l* + 1 denote the indices of the current and next hidden layers, respectively; *j* and *k* represent the indices of neurons in the *l*-th and (*l* + 1)-th layers, respectively; $n_{l}$ indicates the number of neurons in the *l*-th layer; $z_{k}^{\left(l+1\right)}$ is the weighted input of the *k*-th neuron in the (*l* + 1)-th layer; $w_{\mathrm{kj}}^{\left(l+1\right)}$ denotes the connection weight from the *j*-th neuron in the *l*-th layer to the *k*-th neuron in the (*l* + 1)-th layer; $a_{j}^{\left(l\right)}$ and $a_{k}^{\left(l+1\right)}$ are the activation outputs of the *j*-th neuron in the *l*-th layer and the *k*-th neuron in the (*l* + 1)-th layer, respectively; and $b_{k}^{\left(l+1\right)}$ represents the bias term of the *k*-th neuron in the (*l* + 1)-th layer.

The final predicted output voltage value or displacement value of the BP neural network can be denoted by $y_{pred}$:

(4) $$y_{pred}=\sum_{j=1}^{n_{L-1}}w_{1j}^{\left(L\right)}a_{j}^{\left(L-1\right)}+b_{1}^{\left(L\right)}$$

Here, L−1 and L represent the indices of the final hidden layer and the output layer, respectively; $n_{L-1}$ denotes the number of neurons in the final hidden layer; $w_{1j}^{\left(L\right)}$ indicates the connection weight from the *j*-th neuron in the (L−1)-th layer to the first neuron in the output layer; $a_{j}^{\left(L-1\right)}$ is the activation output of the *j*-th neuron in the (L−1)-th layer; and $b_{1}^{\left(L\right)}$ represents the bias term of the first neuron in the output layer.

Meanwhile, in this BP neural network algorithm, the Mean Squared Error (MSE) is used as the loss function:

(5) $$E=\frac{1}{2N}\sum_{n=1}^{N}{\left(y_{true}^{\left(n\right)}-y_{pred}^{\left(n\right)}\right)}^{2}$$

Here, *E* represents the value of the loss function; *N* is the number of training samples; and $y_{true}^{\left(n\right)}$ and $y_{pred}^{\left(n\right)}$ denote the true value and predicted value of the *n*-th sample, respectively.

Finally, the Levenberg–Marquardt algorithm is also employed for weight updating:

(6) $$\Delta w_{ji}^{\left(l\right)}=-\eta \frac{\partial E}{\partial w_{ji}^{\left(l\right)}}=-\eta \delta_{j}^{\left(l\right)}a_{i}^{(l-1)}$$

and bias updating:

(7) $$\Delta b_{j}^{\left(l\right)}=-\eta \frac{\partial E}{\partial b_{j}^{\left(l\right)}}=-\eta \delta_{j}^{\left(l\right)}$$

Here, $\Delta w_{ji}^{\left(l\right)}$ represents the weight update from the *i*-th neuron to the *j*-th neuron in the *l*-th layer; $a_{i}^{(l-1)}$ is the activation output of the *i*-th neuron in the (l−1)-th layer; $\Delta b_{j}^{\left(l\right)}$ and $\delta_{j}^{\left(l\right)}$ denote the bias update and error term of the *j*-th neuron in the *l*-th layer, respectively; and η is the learning rate, which controls the step size for parameter updates.

### 2.2. Data Preparation and Network Construction

#### 2.2.1. Experimental Data Collection and Preprocessing

To evaluate the effectiveness of the BP neural network in predicting the amplitude-frequency response of the Cut-out type piezoelectric vibration energy harvesting system with limiters and assess its generalization performance, the experimental data in this study were divided as follows: First, the gap distance *d* between the Cut-out type piezoelectric beam and the limiter was set to 6 mm and 12 mm, respectively. Then, the installation distance *a* from the limiter to the fixed end of the piezoelectric beam was set to 63 mm, 83 mm, and 103 mm, respectively.

Subsequently, the amplitude–frequency response data of voltage and displacement under forward and reverse frequency sweeps were experimentally measured, yielding six sets of results each. These 12 sets of experimental data were used as training samples to train the BP neural network, enabling it to learn the intrinsic relationship between the distance parameters and the voltage and displacement amplitude–frequency responses in this energy harvesting system.

Finally, the trained BP neural network prediction model was applied to predict the amplitude–frequency responses of voltage and displacement under forward and reverse frequency sweeps for a gap distance *d* of 18 mm and installation distances *a* of 63 mm, 83 mm, and 103 mm, respectively. By comparing the predictions of the BP neural network with the experimentally measured data, the predictive capability of the BP neural network for the voltage and displacement amplitude–frequency responses of this energy harvesting system under unknown distance parameters was directly evaluated, and its reliability in practical applications was validated.

#### 2.2.2. Feature Engineering and Sample Construction

To address the strong nonlinear dynamic characteristics arising from the coupling between the limiters impact mechanism and the structural discontinuity of the Cut-out piezoelectric beam in this energy harvesting system, this study constructs a unified seven-dimensional feature vector *X* that incorporates multi-source information, with the aim of enhancing the representational capacity of the input features. The definitive feature list for both training and inference is defined as

(8) $$X=[d,a,f,\mu_{local},\sigma_{local},\nabla V,\nabla^{2}V]$$

The local features, including $\mu_{local}$, $\sigma_{local}$, gradient ∇V, and curvature $\nabla^{2}V$, are obtained through a sliding window approach with a window width of 20 data points and a step size of 1 data point. During the training phase, these local features are directly extracted from the experimentally measured voltage and displacement response curves. For inference under unseen parameter configurations, the same local features are computed from the preliminary predicted response sequence generated by the BP neural network itself, instead of relying on unavailable experimental data. The sliding window is implemented on the consecutive frequency points of the predicted values to calculate the local statistical indicators and differential geometric features. When dealing with the initial frequency points where less than 19 historical points are accessible, the window is padded with the current predicted value to keep a fixed window width of 20 points.

Since the local features are derived from sparse preliminary predicted samples instead of unknown ground-truth responses, the task of this study is more accurately defined as amplitude–frequency curve reconstruction from sparse predicted samples, rather than pure response prediction.

The sliding window moves with a step size of 1 data point and, for each frequency point, includes the current point and its preceding 19 points; if fewer than 19 points are available before the current point, the window is padded with the value at the current point to maintain a consistent width. Finally, it incorporates two feature parameters extracted based on differential geometric methods: the gradient and curvature of the frequency response curve, which are used to characterize the nonlinear dynamic behavior of this energy harvesting system.

For processing experimental data on the amplitude–frequency response of the Cut-out piezoelectric beam with limiter, a downsampling strategy with an interval of 20 data points is first adopted to extract key experimental data points and establish an initial training sample pool for the BP neural network. Based on this pool, a training dataset consisting of *N* samples is further constructed. This downsampling scheme effectively reduces data volume and accelerates the training efficiency of the BP neural network, while ensuring the downsampled data retain the critical frequency-response information that characterizes the dynamic behavior of the energy harvesting system.

#### 2.2.3. Data Normalization Processing

In order to linearly transform all feature vectors *X* into the [0, 1] interval, the min-max normalization method can be applied for processing:

(9) $$X_{norm}=\frac{X-X_{\min}}{X_{\max}-X_{\min}}$$

Here, $X_{norm}$ represents the normalized feature vector, while *X* is the original feature vector; $X_{\min}$ and $X_{\max}$ denote the minimum and maximum values of the corresponding feature in the training set, respectively. Meanwhile, the output voltage or displacement data are processed using Z-score standardization:

(10) $$Y_{norm}=\frac{Y-\mu_{Y}}{\sigma_{Y}}$$

Here, $Y_{norm}$ represents the standardized voltage or displacement value; Y is the original voltage or displacement value; $\mu_{Y}$ denotes the mean of the voltage or displacement values in the training set; and $\sigma_{Y}$ represents the standard deviation of the voltage or displacement values in the training set.

#### 2.2.4. Neural Network Architecture Design and Optimization

To effectively enhance the training performance and convergence efficiency of this BP neural network, this study adopts the Levenberg–Marquardt algorithm for network training. This algorithm combines the advantages of the gradient descent method and the Gauss–Newton method, accelerating training speed while ensuring convergence stability. In terms of parameters, the maximum training epochs are set to 300, and the target error is set to 1 × 10^−6^, ensuring that the network fully learns while avoiding overfitting and achieving high prediction accuracy.

Additionally, the dataset is partitioned via a strict curve-wise and grouped splitting strategy, rather than a point-wise random split, to mitigate overoptimistic performance estimates induced by strong correlations between adjacent frequency sweep points. Specifically, all experimental data corresponding to limiter gap distances of 6 mm and 12 mm are assigned to the training and validation sets at a 7:1.5 ratio. These data cover installation distances of 63 mm, 83 mm, and 103 mm and include both forward and reverse frequency sweeps. All data from the unseen parameter group with a limiter gap distance of 18 mm are independently used as the test set, contributing 1.5 parts to the overall partitioning ratio. This yields a complete train/validation/test split of 7:1.5:1.5. During training, the validation set is employed for early stopping: training is terminated if the validation loss fails to decrease over 40 consecutive epochs. This grouped splitting approach reserves an entire parameter group and complete frequency sweep curves for testing, adhering to a more rigorous evaluation protocol and preventing data leakage stemming from correlated sweep points. All performance results reported in this study are obtained under this stringent grouped train/validation/test partitioning scheme, guaranteeing the model’s credibility and generalization capability.

**Figure 3:**
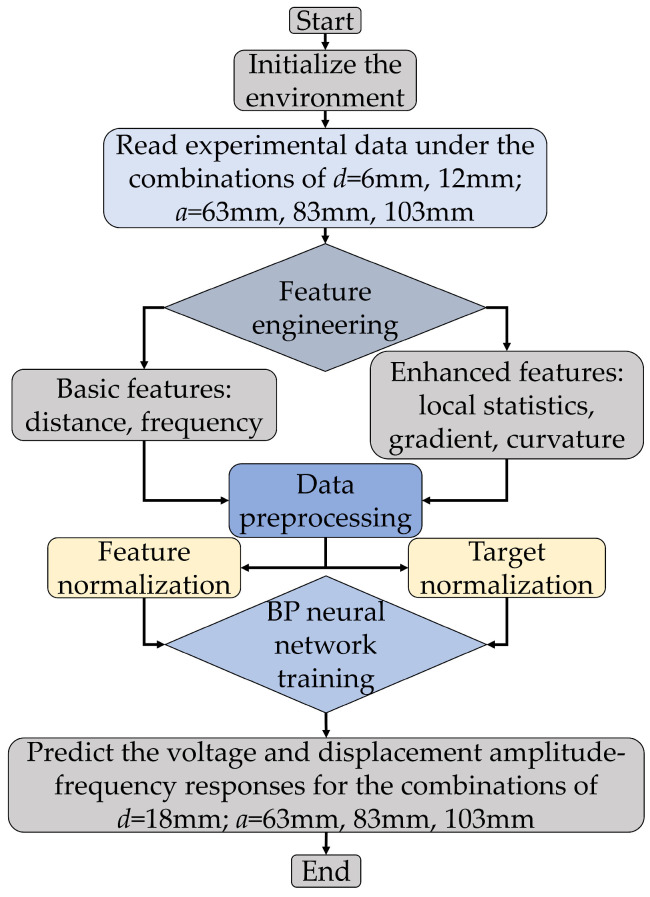
BP neural network algorithm flowchart.

The algorithm flowchart of this BP neural network is shown in Figure 3. By comparing the coefficient of determination *R*^2^ of different network structures on the training set, a large network structure with 50, 30, and 15 nodes is selected as the final configuration. This structure achieves an *R*^2^ value above 0.95 on the training set, striking a good balance between the complexity of the BP neural network and prediction accuracy. The output layer uses a linear activation function to avoid unnecessary constraints on the output range of voltage or displacement values, thereby meeting the requirements of regression tasks.

#### 2.2.5. Prediction Post-Processing and Calibration

To further improve the prediction accuracy of the BP neural network for the voltage and displacement amplitude–frequency responses of the Cut-out piezoelectric energy harvester with limiters, this study proposes a post-processing strategy that relies exclusively on statistics from the training set and the model’s own predictions, ensuring no test-set ground truth is used during inference.

First, to mitigate local discrepancies between model predictions and experimental ground truth in the training set, an exponentially weighted moving average (EWMA) with a fixed weighting factor *α* = 0.85 is adopted to dynamically calibrate the predicted values. The correction coefficients are derived solely from the validation set and then applied to the test-set predictions, which effectively reduces the systematic biases inherent in the BP neural network.

Next, a one-dimensional (1D) Gaussian filter with a window size of 50 data points and standard deviation *σ* = 5 is used to smooth the calibrated frequency–voltage and frequency–displacement sequences. This step effectively suppresses high-frequency fluctuations and random noise, improving the output stability of the BP neural network without introducing any information from the test set.

The applied Gaussian smoothing is mild and data-adaptive, and it only eliminates high-frequency noise and local minor oscillations. Importantly, it does not blur the sharp jump transitions in the nonlinear resonance region caused by the contact-impact of limiters. All key nonlinear dynamic features, including amplitude jumps and frequency hysteresis, are well preserved in the post-processed results.

Through this hierarchical processing framework, which relies solely on the model’s raw outputs and statistics derived from the training set, the reliability and smoothness of the predicted signals are gradually optimized. This ensures that the BP neural network’s predictions of the amplitude–frequency responses for the Cut-out piezoelectric energy harvester with limiters satisfy the dual requirements of stability and accuracy, while maintaining a strict separation between training and test data.

To quantitatively assess the effectiveness of the proposed post-processing strategy without ground-truth fusion, Table 1 compares the prediction performance of the raw BP network output and the post-processed counterpart. All results are obtained under extrapolated parameter settings, where the limiter gap distance is 18 mm and the installation distance is 63 mm. Performance is evaluated using three metrics: *R*^2^, MAE, and RMSE.

As shown in Table 1, post-processed results calibrated via the exponentially weighted moving average (EWMA) method and smoothed using a Gaussian filter yield superior performance relative to raw model outputs. For voltage responses under forward and reverse frequency sweeps, the *R*^2^ values increase from −2.6568 to 0.9888 and from −3.2013 to 0.9943, respectively, with concurrent reductions in both MAE and RMSE. Consistent improvements are also achieved for displacement responses, which exhibit higher *R*^2^ values and correspondingly lower prediction errors.

Notably, no experimental ground truth data are utilized in the post-processing stage, which conforms to the standard protocol of real prediction tasks. The results confirm that the proposed pure data-driven post-processing can effectively enhance the prediction stability and accuracy without introducing unavailable information during inference, thus avoiding overestimation of the model performance.

## 3. Experimental Setup and Methodology

### 3.1. Structure of the Cut-Out Type Energy Harvester with Limiters

**Figure 4:**
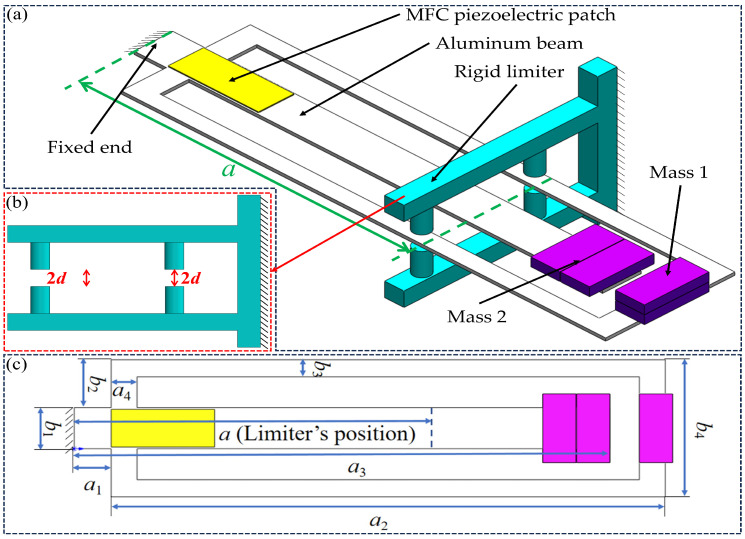
Schematic of a Cut-out piezoelectric beam with motion limiter configuration: (**a**) nonlinear configuration with limiters; (**b**) two pairs of bilateral symmetric limiters; (**c**) Cut-out beam [37].

This paper predicts the voltage and displacement amplitude–frequency responses of a novel piezoelectric vibration energy harvesting structure. The energy harvester consists of a Cut-out type piezoelectric beam, displacement limiters, an end-mounted concentrated mass, and a base, as shown in Figure 4. The material and geometric parameters of the Cut-out beam and the piezoelectric patch are listed in Table 2.

The piezoelectric beam consists of an outer frame-type main beam and a cantilever-type auxiliary beam, both with free ends equipped with additional masses. A Macro Fiber Composite (MFC) piezoelectric patch is attached near the fixed-end connection, so that their vibrations jointly affect the voltage output. Two pairs of displacement limiters are arranged symmetrically above and below on both sides of the main beam.

### 3.2. Experimental Facility

**Figure 5:**
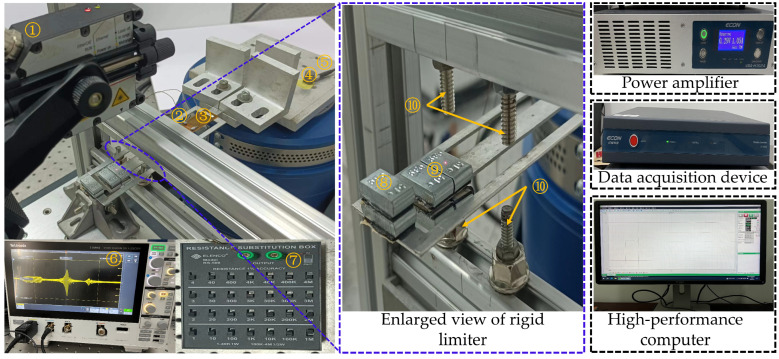
Schematics of experimental setup [37]: ① laser displacement sensor; ② piezoelectric aluminum beam; ③ MFC piezoelectric patch; ④ acceleration sensor; ⑤ vibration table; ⑥ oscilloscope; ⑦ external resistor; ⑧ mass 1; ⑨ mass 2; ⑩ rigid limiter.

The experimental facility is illustrated in Figure 5. The root of the Cut-out piezoelectric beam is fixed to a vibration table driven by an ECON vibration system controlled via a high-performance computer. The ECON vibration system has an effective frequency range of 5–5000 Hz, and the power amplifier is set to a constant value of 66% for this experiment. Longitudinal harmonic vibration is induced by controlling the accelerometer to drive the vibration table.

### 3.3. Experimental Data Acquisition

The experimental design is grouped based on two distance parameters, *d* and *a*: the gap distance *d* between the piezoelectric beam and the limiter is set to three groups—6 mm, 12 mm, and 18 mm. Within each group, the distance *a* from the limiter to the fixed end of the piezoelectric beam is further divided into three subgroups: 63 mm, 83 mm, and 103 mm. Forward and reverse frequency sweep experiments are performed for each subgroup.

To ensure the reliability and repeatability of the experimental data, three repeated data acquisitions are conducted for each experimental group under specific distance parameters *d* and *a* and sweep direction. The coefficient of variation (*C_V_*), calculated as the standard deviation divided by the mean and multiplied by 100%, is computed for the displacement amplitude and voltage amplitude at each frequency point. It is required that the *C_V_* for all frequency points does not exceed 5%. If the *C_V_* for any frequency point exceeds this threshold, the entire experiment for that group is repeated until the *C_V_* for all critical frequency points meets the requirement.

Finally, the experimental data for each group is averaged over three valid repetitions and serves as the basis for subsequent amplitude–frequency response analysis and data-driven modeling with the BP neural network.

## 4. Prediction Results and Discussion

### 4.1. Comparative Analysis of Predicted Response and Experimental Results

**Figure 6:**
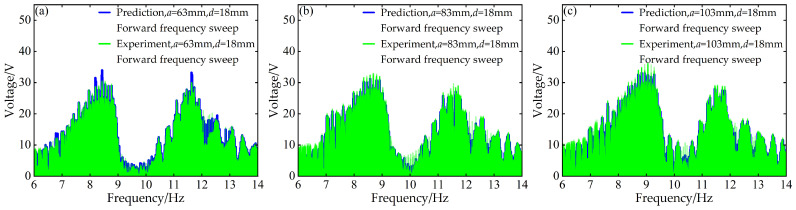
Comparison between predicted and experimental voltage amplitude–frequency responses at *d* = 18 mm during forward frequency sweep: (**a**) *a* = 63 mm; (**b**) *a* = 83 mm; (**c**) *a* = 103 mm.

**Figure 7:**
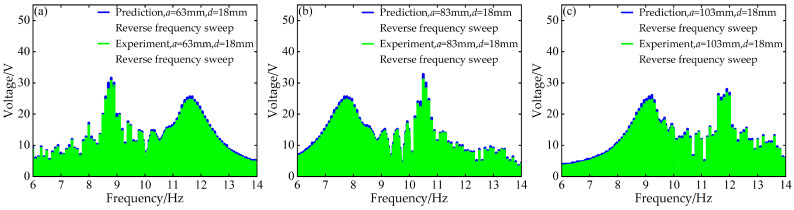
Comparison between predicted and experimental voltage amplitude–frequency responses at *d* = 18 mm during reverse frequency sweep: (**a**) *a* = 63 mm; (**b**) *a* = 83 mm; (**c**) *a* = 103 mm.

**Figure 8:**
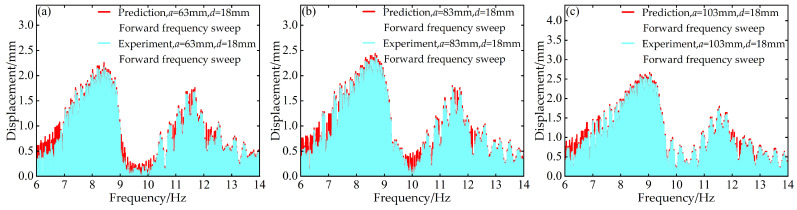
Comparison between predicted and experimental displacement amplitude–frequency responses at *d* = 18 mm during forward frequency sweep: (**a**) *a* = 63 mm; (**b**) *a* = 83 mm; (**c**) *a* = 103 mm.

**Figure 9:**
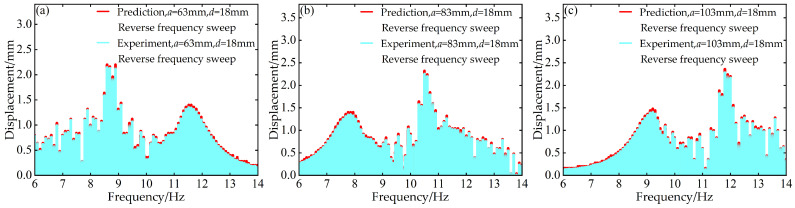
Comparison between predicted and experimental displacement amplitude–frequency responses at *d* = 18 mm during reverse frequency sweep: (**a**) *a* = 63 mm; (**b**) *a* = 83 mm; (**c**) *a* = 103 mm.

To validate the modeling capability of the BP neural network for the nonlinear dynamic behavior of the Cut-out type piezoelectric vibration energy harvesting system with limiters, this study uses experimental data at *d* = 6 mm and *d* = 12 mm with distance parameter *a* values of 63 mm, 83 mm, and 103 mm to train the BP neural network. Based on this, the voltage and displacement amplitude–frequency responses at *d* = 18 mm for the corresponding *a* values are predicted. A comparison between the prediction results and the experimental data is shown in Figure 6, Figure 7, Figure 8 and Figure 9.

At *d* = 18 mm and *a* = 63 mm, for both forward and reverse frequency sweeps, the coefficient of determination *R*^2^ for the predicted voltage amplitude–frequency responses reached 0.988842 and 0.994329, respectively. The mean absolute error (MAE) was 0.195959 V and 0.186913 V, while the root mean square error (RMSE) was 0.483112 V and 0.296438 V, respectively. For the displacement amplitude–frequency responses, the *R*^2^ values were as high as 0.995078 and 0.998393, with MAE values of 0.011379 mm and 0.008037 mm and RMSE values of 0.025029 mm and 0.012310 mm, respectively.

Under the distance parameters of *d* = 18 mm and *a* = 83 mm, the *R*^2^ values for the voltage and displacement amplitude–frequency responses during forward frequency sweeps reached 0.990166 and 0.995068, respectively, with MAE values of 0.221633 V and 0.012361 mm and RMSE values of 0.496684 V and 0.027122 mm. During reverse frequency sweeps, the *R*^2^ values reached 0.994728 and 0.998380, with MAE values of 0.183859 V and 0.008015 mm and RMSE values of 0.286433 V and 0.012338 mm, respectively.

Additionally, when the distance parameter *a* takes the maximum value of 103 mm, the BP neural network prediction model still demonstrates good prediction accuracy for the amplitude–frequency responses of voltage and displacement. Specifically, during forward frequency sweeps, the *R*^2^ values are as high as 0.991780 and 0.995279, respectively, while during reverse frequency sweeps, they further improve to 0.994266 and 0.998490. The corresponding error metrics also remain at low levels: during forward sweeps, the MAE values are 0.219819 V and 0.012657 mm, and the RMSE values are 0.474050 V and 0.028608 mm; during reverse sweeps, the MAE values are 0.180013 V and 0.007546 mm, and the RMSE values are 0.292947 V and 0.012031 mm.

The above results indicate that the BP neural network is capable of predicting the nonlinear dynamic responses of the system with high accuracy in most cases, demonstrating strong generalization capability.

**Figure 10:**
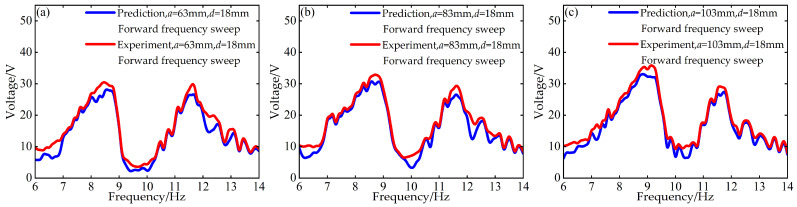
Comparison between predicted and experimental outer envelopes of voltage amplitude–frequency responses at *d* = 18 mm during forward frequency sweep: (**a**) *a* = 63 mm; (**b**) *a* = 83 mm; (**c**) *a* = 103 mm.

**Figure 11:**
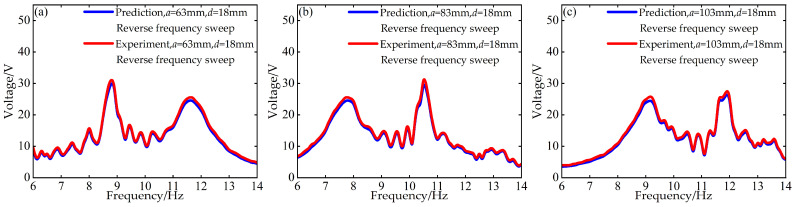
Comparison between predicted and experimental outer envelopes of voltage amplitude–frequency responses at *d* = 18 mm during reverse frequency sweep: (**a**) *a* = 63 mm; (**b**) *a* = 83 mm; (**c**) *a* = 103 mm.

**Figure 12:**
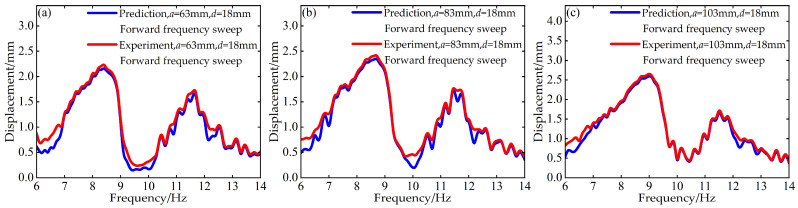
Comparison between predicted and experimental outer envelopes of displacement amplitude–frequency responses at *d* = 18 mm during forward frequency sweep: (**a**) *a* = 63 mm; (**b**) *a* = 83 mm; (**c**) *a* = 103 mm.

**Figure 13:**
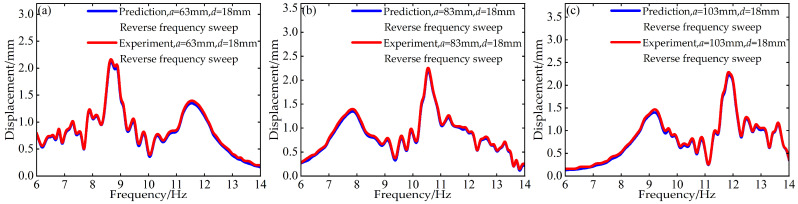
Comparison between predicted and experimental outer envelopes of displacement amplitude–frequency responses at *d* = 18 mm during reverse frequency sweep: (**a**) *a* = 63 mm; (**b**) *a* = 83 mm; (**c**) *a* = 103 mm.

To more clearly reveal the differences between the amplitude–frequency response results predicted by the BP neural network and the experimental data, particularly the characteristics of response amplitude and bandwidth in the nonlinear resonance region of the energy harvesting system, this study further plots the envelope curves of the voltage and displacement amplitude–frequency responses under different operating conditions, as shown in Figure 10, Figure 11, Figure 12 and Figure 13.

In the view of the envelope curves, the predictive performance of the BP neural network model can be more intuitively demonstrated. Overall, under different distance parameters *d* and *a*, the envelope curves of the amplitude–frequency responses predicted by the BP neural network align well with those measured experimentally. This indicates that the BP neural network not only accurately reproduces the peak responses of the energy harvesting system but also successfully captures the bandwidth broadening and hysteresis characteristics caused by the strong nonlinearity of the Cut-out type piezoelectric vibration energy harvesting system with limiters.

Notably, as shown in Figure 11 and Figure 13, under reverse frequency sweep conditions, regardless of whether the distance parameter *d* is set to 18 mm, 83 mm, or 103 mm, the envelope curves of the amplitude–frequency responses predicted by the BP neural network almost completely overlap with those measured experimentally. This visually verifies the high coefficient of determination *R*^2^ (greater than 0.990) and the low error metrics (MAE and RMSE) observed in the earlier predictions of displacement amplitude–frequency responses during reverse sweeps. This consistently demonstrates that the BP neural network prediction model exhibits good prediction accuracy and reliability within the frequency range of 6~14 Hz.

Furthermore, these results not only indicate that the BP neural network prediction model delivers strong predictive performance across various distance parameter combinations, highlighting its favorable generalization capability and stability, but also confirm the feasibility of the data-driven modeling approach based on BP neural networks for the modeling and analysis of strongly nonlinear piezoelectric vibration energy harvesting systems.

### 4.2. Model Performance Evaluation

To investigate the persistent discrepancy in prediction accuracy between forward and reverse frequency sweeps, a concise diagnostic analysis is carried out by plotting the absolute voltage prediction error (AVPE) and absolute displacement prediction error (ADPE) as functions of excitation frequency. Results indicate that the absolute errors from reverse-sweep predictions are almost consistently lower than those from forward-sweep predictions, and the predicted response envelope curves for reverse sweeps also align nearly perfectly with experimental measurements.

**Figure 14:**
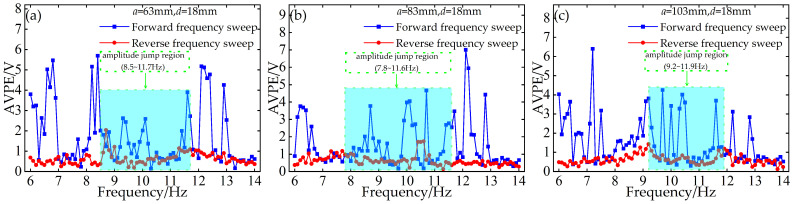
Absolute voltage prediction error vs. frequency for forward and reverse frequency sweeps: (**a**) *a* = 63 mm, *d* = 18 mm; (**b**) *a* = 83 mm, *d* = 18 mm; (**c**) *a* = 103 mm, *d* = 18 mm.

**Figure 15:**
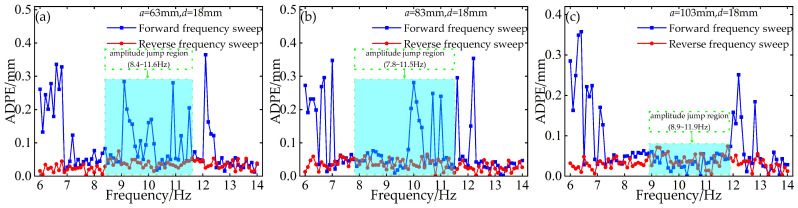
Absolute displacement prediction error vs. frequency for forward and reverse frequency sweeps: (**a**) *a* = 63 mm, *d* = 18 mm; (**b**) *a* = 83 mm, *d* = 18 mm; (**c**) *a* = 103 mm, *d* = 18 mm.

Tests were performed with a limiter gap distance *d* = 18 mm and installation distances *a* of 63 mm, 83 mm, and 103 mm, respectively. Figure 14 presents the AVPE under forward and reverse sweeps, and Figure 15 shows the corresponding ADPE under identical test conditions. The light blue shaded regions in Figure 14 and Figure 15 denote the nonlinear resonance band, where amplitude jumps and hysteretic behavior occurs.

It is evident that reverse-sweep prediction errors remain consistently lower across all test conditions, particularly within the frequency band where the nonlinear system exhibits abrupt amplitude jumps and hysteresis. This discrepancy stems directly from the inherent hysteresis and jump dynamics of contact-impact nonlinear systems. During forward frequency sweeping, the system undergoes an abrupt transition from low-amplitude to high-amplitude oscillations at the jump frequency, accompanied by intense transient fluctuations and heightened nonlinear uncertainty. By contrast, during reverse sweeping, the system sustains a relatively stable high-energy response branch prior to the downward transition, resulting in smoother dynamic evolution and more regular response patterns. Such well-behaved dynamics can be captured more effectively by the BP neural network, thus yielding smaller prediction errors and higher overall predictive accuracy.

This diagnostic plot of absolute error versus excitation frequency confirms that the superior predictive performance of reverse sweeps is fundamentally linked to the inherent hysteretic and jump characteristics of the contact-impact nonlinear system.

**Figure 16:**
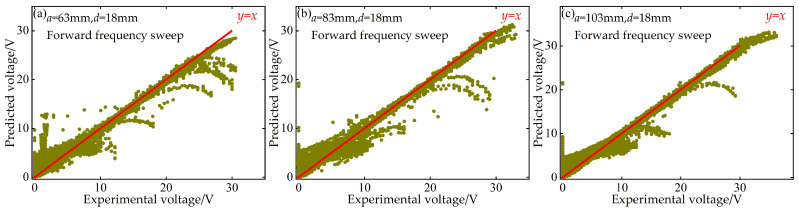
Scatter plot of predicted versus experimental voltage data at *d* = 18 mm during forward frequency sweep: (**a**) *a* = 63 mm; (**b**) *a* = 83 mm; (**c**) *a* = 103 mm.

**Figure 17:**
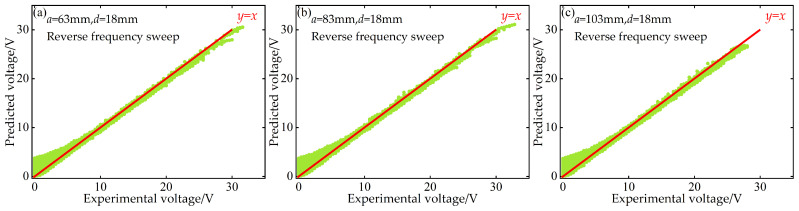
Scatter plot of predicted versus experimental voltage data at *d* = 18 mm during reverse frequency sweep: (**a**) *a* = 63 mm; (**b**) *a* = 83 mm; (**c**) *a* = 103 mm.

**Figure 18:**
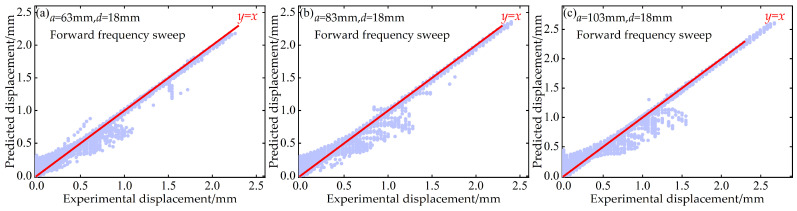
Scatter plot of predicted versus experimental displacement data at *d* = 18 mm during forward frequency sweep: (**a**) *a* = 63 mm; (**b**) *a* = 83 mm; (**c**) *a* = 103 mm.

**Figure 19:**
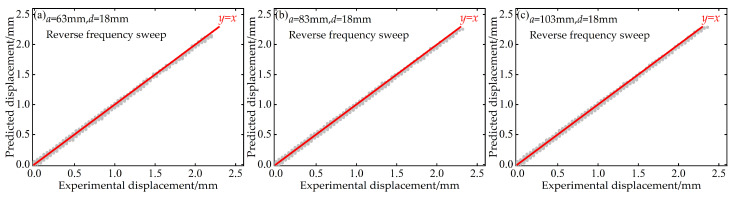
Scatter plot of predicted versus experimental displacement data at *d* = 18 mm during reverse frequency sweep: (**a**) *a* = 63 mm; (**b**) *a* = 83 mm; (**c**) *a* = 103 mm.

Additionally, to quantitatively evaluate the predictive performance of the BP neural network at the “point-to-point” accuracy level, this paper also plots scatter diagrams of voltage and displacement between the predicted values of the BP neural network and the experimentally measured values during both forward and reverse frequency sweeps. These diagrams are supplemented with the ideal prediction line *y* = *x* as a benchmark, as shown in Figure 16, Figure 17, Figure 18 and Figure 19.

These scatter diagrams provide a microscopic perspective on the predictive performance of the BP neural network. Ideally, all data points should be evenly distributed along the diagonal line *y* = *x*. The results indicate that under different distance parameter combinations, the vast majority of data points are closely clustered around the *y* = *x* diagonal line, confirming the high predictive accuracy of the BP neural network.

**Figure 20:**
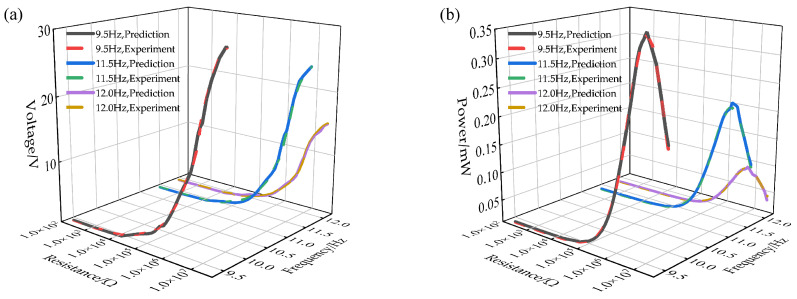
Comparison between predicted and experimental data at *a* = 63 mm and *d* = 6 mm under different frequencies: (**a**) resistance–voltage data; (**b**) resistance–power data.

Finally, to further validate the practical value of the constructed BP neural network prediction model in optimizing the output performance of nonlinear vibration piezoelectric energy harvesting systems, this paper analyzes the resistance-voltage and resistance-power curves at three characteristic frequencies—9.5 Hz, 11.5 Hz, and 12.0 Hz—based on the selected distance parameters *a* = 63 mm and *d* = 6 mm. A comparison between the prediction results and the experimental data is shown in Figure 20. The results demonstrate that the predicted load resistance–output voltage and load resistance–output power curves exhibit a high degree of consistency with the experimental data.

**Figure 21:**
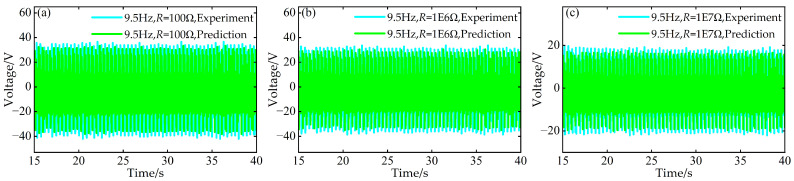
Comparison between predicted and experimental time–voltage response curves at *a* = 63 mm, *d* = 6 mm, and 9.5 Hz under different resistances: (**a**) 100 Ω; (**b**) 1 × 10^6^ Ω; (**c**) 1 × 10^7^ Ω.

**Figure 22:**
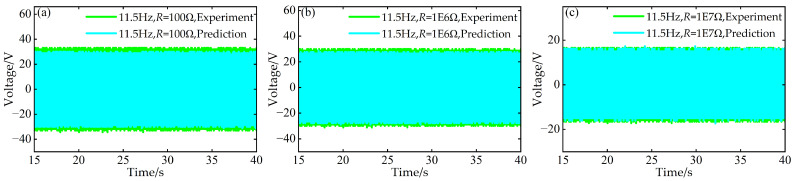
Comparison between predicted and experimental time–voltage response curves at *a* = 63 mm, *d* = 6 mm, and 11.5 Hz under different resistances: (**a**) 100 Ω; (**b**) 1 × 10^6^ Ω; (**c**) 1 × 10^7^ Ω.

**Figure 23:**
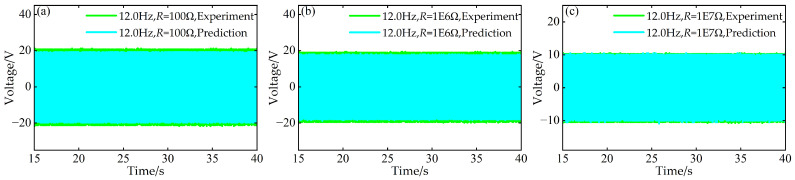
Comparison between predicted and experimental time–voltage response curves at *a* = 63 mm, *d* = 6 mm, and 12.0 Hz under different resistances: (**a**) 100 Ω; (**b**) 1 × 10^6^ Ω; (**c**) 1 × 10^7^ Ω.

Simultaneously, at the aforementioned frequencies of 9.5 Hz, 11.5 Hz, and 12.0 Hz, using experimental data for time–voltage responses under resistances of *R* = 1 × 10^3^ Ω, 1 × 10^4^ Ω, and 1 × 10^5^ Ω, predictions were made for the time–voltage responses under three different external resistance values: *R* = 1 × 10^2^ Ω, 1 × 10^6^ Ω, and 1 × 10^7^ Ω. A comparative diagram is shown in Figure 21, Figure 22 and Figure 23.

**Figure 24:**
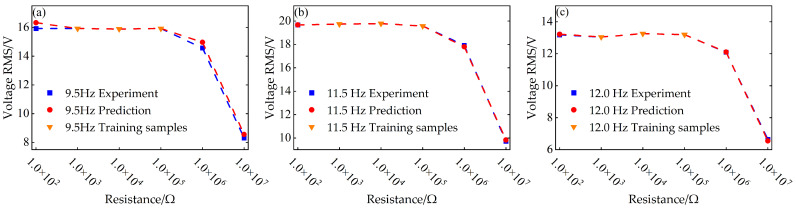
Comparison of resistance–voltage RMS curves between predicted results and experimental data at different frequencies when *a* = 63 mm and *d* = 6 mm: (**a**) 9.5 Hz; (**b**) 11.5 Hz; (**c**) 12.0 Hz.

Among these, at 9.5 Hz and *R* = 1 × 10^7^ Ω, the root mean square (RMS) value of the predicted result differed from the RMS value of the experimental data by only 0.2499 V. At 11.5 Hz and *R* = 1 × 10^2^ Ω, the difference between the RMS values of the predicted result and the experimental data further decreased to 0.0057 V. When the frequency increased to 12.0 Hz, the difference between the predicted result and the experimental data at *R* = 1 × 10^6^ Ω was still only 0.0189 V. A comparison diagram of the predicted and experimental results for resistance and voltage RMS values is shown in Figure 24.

By validating the resistance–voltage and resistance–power relationships across multiple frequency points, this study fully demonstrates, from the perspective of system optimization design, that the BP neural network prediction model can accurately predict the external characteristics of nonlinear vibration piezoelectric energy harvesting systems under different resistive loads. This, in turn, provides a theoretical basis and model foundation for subsequent circuit design and the development of energy management strategies.

### 4.3. Baseline Model Comparison

To further verify the superiority of the proposed multi-layer BP neural network integrated with feature engineering and post-processing calibration, two lightweight baseline models are implemented under the same evaluation protocol for quantitative comparison.

The first baseline is Gaussian Process Regression (GPR), used as a conventional non-parametric fitting benchmark to represent classic data-driven modeling methods for nonlinear dynamic systems. The second baseline is Support Vector Regression (SVR), a typical lightweight machine learning model with kernel-based nonlinear mapping capability. It adopts the same dataset partitioning strategy as the proposed model, but does not involve deep multi-layer architecture, dedicated feature engineering, or post-processing calibration modules.

Both baselines were trained and validated on the same dataset with a strict grouped train–validation–test split ratio of 7:1.5:1.5. Testing was performed on the same extrapolated parameter dataset as the proposed BP network, corresponding to the limiter gap distance *d* = 18 mm and installation distance *a* = 63 mm.

**Figure 25:**
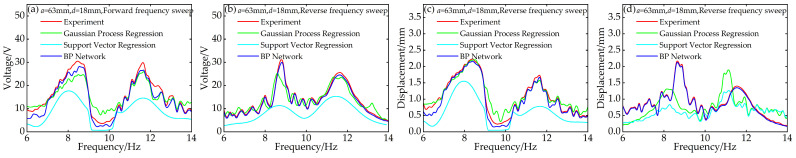
Under the condition of *d* = 18 mm and *a* = 63 mm, comparison of experimental measurements with predictions from GPR, SVR, and BP Network for (**a**) forward voltage sweep, (**b**) reverse voltage sweep, (**c**) forward displacement sweep, and (**d**) reverse displacement sweep.

Quantitative comparison results are summarized in Table 3. Figure 25 presents the comparison curves of the experimental measurement results and the three models, namely GPR, SVR, and BP Network, for the voltage and displacement responses under forward and reverse frequency sweeps.

Among the three types of models, the SVR model exhibits the most limited overall prediction performance. Although it achieves a certain degree of nonlinear fitting by leveraging kernel functions, it is constrained by the lack of deep feature learning and post-processing calibration, making it difficult to fully capture the strongly nonlinear behavior of the energy harvesting system. The GPR model shows improved representational capability; however, its prediction accuracy remains inadequate in the resonance region characterized by severe amplitude fluctuations and hysteresis, manifesting as a low *R*^2^ value and significant prediction errors. In contrast, the multilayer BP neural network incorporating feature engineering and post-processing calibration, as constructed in this study, consistently outperforms the aforementioned baseline models across all evaluation metrics (*R*^2^, MAE, and RMSE) in predicting the voltage and displacement amplitude-frequency responses under both forward and backward sweeping conditions. These results indicate that the combination of "multilayer network architecture, targeted feature engineering, and data-driven post-processing calibration" collectively contributes to a significant enhancement in the predictive performance for this strongly nonlinear piezoelectric vibration energy harvesting system, rendering the proposed BP neural network the best-performing method among the three approaches.

## 5. Conclusions

This study addresses the challenges of modeling the strong nonlinearity caused by mechanical contact-impact in Cut-out type piezoelectric beams with limiters, as well as the high cost associated with full parametric domain experimental measurements. A data-driven modeling method based on BP neural networks is proposed. Through systematic experiments and predictive analysis, the applicability and accuracy of this method in modeling strongly nonlinear piezoelectric vibration energy harvesting systems have been validated. The main conclusions are as follows:

(1) The prediction model constructed using the BP neural network can effectively predict the amplitude–frequency responses of voltage and displacement under the extrapolated distance parameter *d* = 18 mm, relying on experimental data from limited distance parameter combinations (*d* = 6 mm and *d* = 12 mm). This overcomes the modeling bottleneck of traditional mechanistic models in dealing with the strong nonlinearity introduced by mechanical impact.
(2) Under both forward and reverse frequency sweep excitations, the coefficient of determination *R*^2^ for the predicted voltage and displacement amplitude–frequency responses of the BP neural network model is consistently above 0.980. The maximum mean absolute errors are 0.222 V and 0.013 mm, respectively, while the maximum root mean square errors are 0.4967 V and 0.029 mm, respectively. Furthermore, the model’s accurate prediction of transient voltage responses under different load resistances *R* further validates its strong generalization capability.
(3) The data-driven modeling approach based on BP neural networks significantly reduces reliance on physical experiments and avoids the extensive testing required to obtain performance maps across the full parameter domain. This provides an efficient analytical method for the rapid performance evaluation and structural optimization of strongly nonlinear piezoelectric vibration energy harvesters. Future research will incorporate advanced strategies such as ensemble learning, aiming to enhance the predictive stability and generalization of the BP neural network in unseen operational scenarios, such as novel structural parameters or unknown excitation levels, through the construction of multi-model consensus mechanisms.

## Figures and Tables

**Table 1:** Performance comparison between raw BP network output and post-processed output.

Response Type	Post-Processing Stage	*R* ^2^	MAE	RMSE
Voltage (Forward sweep)	Raw model output	−2.6568	8.1088 V	8.7459 V
	Post-processed output	0.9888	0.1960 V	0.4831 V
Voltage (Reverse sweep)	Raw model output	−3.2013	7.3801 V	8.0685 V
	Post-processed output	0.9943	0.1869 V	0.2964 V
Displacement (Forward sweep)	Raw model output	−4.9141	0.8445 mm	0.8676 mm
	Post-processed output	0.9951	0.0114 mm	0.0250 mm
Displacement (Reverse sweep)	Raw model output	−6.3139	0.8120 mm	0.8304 mm
	Post-processed output	0.9984	0.0080 mm	0.0123 mm

**Table 2:** Material and geometric parameters.

Parameter	Value
**Beam**	
Young’s modulus (*E_s_*_1_)	70 GPa
Mass density (*ρ_s_*_1_)	2700 kg m^−3^
Length (*a*_1_)	10 mm
Length (*a*_2_)	150 mm
Length (*a*_3_)	145 mm
Length (*a*_4_)	7 mm
Width (*b*_1_)	12 mm
Width (*b*_2_)	14 mm
Width (*b*_3_)	5 mm
Width (*b*_4_)	40 mm
Thickness	1 mm
**Piezoelectric member**	
Length	20 mm
Width	10 mm
Thickness	0.3 mm
Piezoelectric constant	670 C/N
**Proof mass**	
Mass1	17 g
Mass2	19 g

**Table 3:** Performance comparison between the proposed BP network and baseline models.

Model	Response Type	Sweep Direction	*R* ^2^	MAE	RMSE
GPR	Voltage	Forward sweep	0.8433	1.3600 V	1.8104 V
		Reverse sweep	0.9297	0.5626 V	1.0436 V
	Displacement	Forward sweep	0.9069	0.0700 mm	0.1089 mm
		Reverse sweep	0.7639	0.0760 mm	0.1492 mm
SVR	Voltage	Forward sweep	0.6786	1.2897 V	2.5929 V
		Reverse sweep	0.6921	1.0420 V	2.1844 V
	Displacement	Forward sweep	0.6879	0.0970 mm	0.1993 mm
		Reverse sweep	0.6673	0.1133 mm	0.1771 mm
BP Network	Voltage	Forward sweep	0.9888	0.1960 V	0.4831 V
		Reverse sweep	0.9943	0.1869 V	0.2964 V
	Displacement	Forward sweep	0.9951	0.0114 mm	0.0250 mm
		Reverse sweep	0.9984	0.0080 mm	0.0123 mm

## Data Availability

The raw data supporting the conclusions of this article will be made available by the authors on request.
